# Structural and Hemodynamic Analyses of Different Stent Structures in Curved and Stenotic Coronary Artery

**DOI:** 10.3389/fbioe.2019.00366

**Published:** 2019-12-06

**Authors:** Lingling Wei, Hwa Liang Leo, Qiang Chen, Zhiyong Li

**Affiliations:** ^1^Biomechanics Laboratory, School of Biological Science & Medical Engineering, Southeast University, Nanjing, China; ^2^Department of Biomedical Engineering, National University of Singapore, Singapore, Singapore; ^3^School of Chemistry, Physics and Mechanical Engineering, Queensland University of Technology, Brisbane, QLD, Australia

**Keywords:** coronary artery stenting, stent structure, in-stent restenosis, structural mechanics, hemodynamics

## Abstract

Coronary artery stenting is commonly used for the treatment of coronary stenosis, and different stent structures indeed have various impacts on the stress distribution within the plaque and artery as well as the local hemodynamic environment. This study aims to evaluate the performance of different stent structures by characterizing the mechanical parameters after coronary stenting. Six stent structures including three commercially-shaped stents (Palmaz-Schatz-shaped, Xience Prime-shaped, and Cypher-shaped) and three author-developed stents (C-Rlink, C-Rcrown, and C-Astrut) implanted into a curved stenotic coronary artery were investigated. Structural analyses of the balloon-stent-plaque-artery system were first performed, and then followed by hemodynamic analyses. The results showed that among the three commercially-shaped stents, the Palmaz-Schatz-shaped had the least stent dogboning and recoiling, corresponding to the greatest maximum plastic strain and the largest diameter change, nevertheless, it induced the highest maximum von Mises stress on plaque, arterial intima and media. From the viewpoint of hemodynamics, the Palmaz-Schatz-shaped displayed smaller areas of adverse low wall shear stress (<0.5 Pa), low time-averaged wall shear stress (<0.5 Pa), and high oscillating shear index (>0.1). Compared to the Cypher-shaped, the C-Rcrown and C-Astrut had smaller recoiling, greater maximum plastic stain and larger diameter change, which indicated the improved mechanical performance of the Cypher-shaped stent. Moreover, both C-Rcrown and C-Astrut exhibited smaller areas of adverse low wall shear stress, and low time-averaged wall shear stress, but only the C-Rcrown displayed a smaller area of adverse high oscillating shear index. The present study evaluated and compared the performance of six different stents deployed inside a curved artery, and could be potentially utilized as a guide for the selection of suitable commercially-shaped stent for clinical application, and to provide an approach to improve the performance of the commercial stents.

## Introduction

Coronary artery disease (CAD) is one of the greatest threats to human health and life, and it usually occurs when cholesterol builds up inside the walls of arteries, eventually forming a plaque, thus narrowing the arteries and limiting the flow of oxygen-rich blood to the heart. At present, percutaneous transluminal coronary angioplasty is one of the main methods for the treatment of CAD, owing to its advantages of little surgical trauma, short treatment time and quick rehabilitation (Mueller and Sanborn, 1995). Among the percutaneous interventions, stenting is the most common procedure to treat coronary stenosis. However, serious clinical complications remain such as the in-stent restenosis (ISR), which is the reduction of the lumen size following the stent implantation (Park et al., 2012).

The primary process leading to ISR is neointimal hyperplasia (NH) that consists of excessive tissue growth in and around the implanted stent, and results in a decreased blood flow through the artery. On the one hand, mechanical stress is exerted on plaque and artery with the presence of the stent, causing injury to the plaque and artery, and further promoting ISR (Timmins et al., 2011). On the other hand, the local hemodynamic environment is altered as well, which causes abnormal shear stress on the endothelial cells (Wentzel et al., 2008). Sites exposed to low wall shear stress (WSS) and high low oscillating shear index (OSI) are particularly susceptible to the development of atherosclerotic tissue and intimal thickening, which leads to ISR (Ku et al., 1985; He and Ku, 1996; Buchanan et al., 2003). It is reported that different stent structures not only caused varying stress levels within the plaque and artery (Colombo et al., 2002; Gu et al., 2010), but also induced different levels and patterns of WSS and OSI on the artery wall (Balossino et al., 2008; Murphy and Boyle, 2010a; Pant et al., 2010; Gundert et al., 2013), so the stent structure plays a critical role in the post-operative effect of stenting. The structure of the stent cell is the focus of many studies, and it is shown that patients treated with closed-cell stents had a lower risk of experiencing adverse events compared to those treated with open-cell stents (Hart et al., 2006), and the rates of the post-operative complication were also higher for the open-cell stents, especially in symptomatic patients, which increased with a larger free cell area (Bosiers et al., 2008). Computational optimization on the number of strut crowns revealed that the optimal number was dependent on the intra-strut angle with respect to the blood flow direction (Gundert et al., 2012b). Besides, different connection methods of struts such as peak-to-peak (aligned stent strut) or peak-to-valley (offset stent strut) also influenced the blood flow (Gundert et al., 2012a; Beier et al., 2016).

The finite element analysis (FEA) method has been widely used to evaluate the biomechanical performance of the stent, the contact between stent and balloon, and the stent-plaque-artery interaction. To study the factors that affect the ISR and to optimize the stent structure, a number of structural analyses involving stent deployment in the artery were investigated (Lally et al., 2005; Wang et al., 2006; Wu et al., 2007; Conti et al., 2011; Pant et al., 2012; Bukala et al., 2017; Liu et al., 2018; Shen et al., 2018), as well as many computational fluid dynamic (CFD) researches studying the local hemodynamics of stented arteries (Balossino et al., 2008; Murphy and Boyle, 2010b; Morlacchi et al., 2011b; Rikhtegar et al., 2013, 2014; Beier et al., 2016; Foucault et al., 2017; Yu et al., 2017). However, in the majority of these studies, both the stent and the lumen geometries were assumed to be ideally non-deformed. These idealized models neglected the complex features of the deformed stent and lumen, which had a major effect on the hemodynamic environment in stented coronary arteries (Martin et al., 2014). Besides, most of these studies only evaluated stent performance from the viewpoint of structural mechanics or hemodynamics, and the assessment from the combined two aspects has not been fully studied. Since Morlacchi et al. (2011a) first successfully introduced sequential structural and fluid dynamic simulations of stent deployment in coronary bifurcations, the combined method of the structural and hemodynamic analyses was employed as an effective approach to investigate the post-stenting effect on the blood flow. For example, it was used to study the post-stenting hemodynamics to optimize fluid dynamic simulation method (Chiastra et al., 2012), to guide clinical applications (Mortier et al., 2015), and to better study the clinical stenting technique (Morris et al., 2018).

To this end, both structural and hemodynamic analyses were here performed to evaluate the performance of different stent structures. Six stents including three commercially-shaped stents, Palmaz-Schatz-shaped (PS-shaped), Xience Prime-shaped (XP-shaped), and Cypher-shaped (C-shaped), and three author-developed stents, C-Rlink, C-Rcrown, and C-Astrut were constructed separately. Addressing the six stents, structural analyses were firstly performed to obtain the deformed luminal boundaries of a curved artery. On the basis of the luminal boundaries, hemodynamic analyses were then conducted to quantify the critical hemodynamic parameters. Moreover, the effects of stent structure by changing the structure and connection way of the stent struts were investigated. The present study will provide a better understanding of the deployment of different stents inside curved stenotic arteries to facilitate the clinical choice of suitable commercial stents, also be helpful to the design of the stent from the perspective of the structure and connection of stent struts.

## Materials and Methods

### Geometric Models

#### Stent

**Figure 1:**
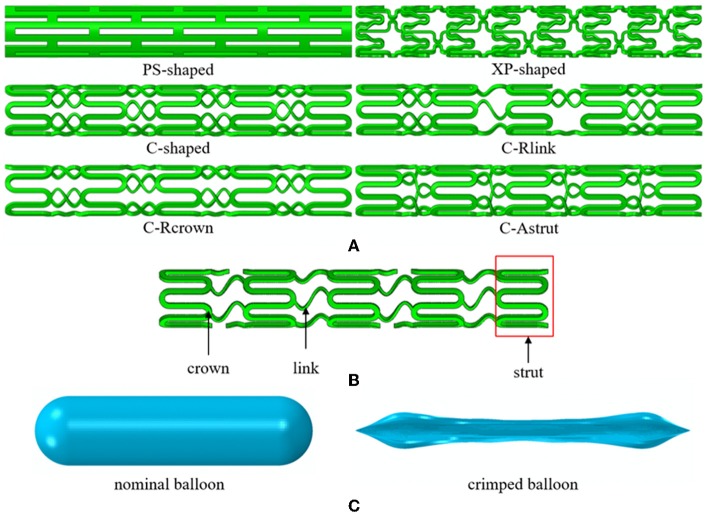
**(A)** The geometries for six stent structures; **(B)** the C-shaped stent geometry with stent design labels; **(C)** the nominal and crimped geometries of the balloon.

The previous study showed that stent with S-type link (similar to commercial Cypher stent) performed better in arteries with different curvatures (Wei et al., 2016). To verify whether stent performance could be improved by the modification of the number of links, the number of crowns, or the connection method of struts, the C-shaped stent with a reduced number of links (C-Rlink), C-shaped stent with a reduced number of crowns (C-Rcrown), and C-shaped stent with aligned struts (C-Astrut) were developed, respectively. The three author-developed stents together with three commercially-shaped stents (PS-shaped, XP-shaped, and C-shaped) were shown in Figures 1A,B, and the length, the external diameter at crimped state, and the radial thickness of all stents were set as 10.0, 1.5, and 0.1 mm, respectively. Note that the real commercial Palmaz-Schatz, Xience Prime, and Cypher stents had different strut thicknesses, while the strut thicknesses of the six stents here were identical in order to compare the biomechanical effects of different stent structures by eliminating the strut-thickness factor.

#### Balloon

The balloon was designed with a nominal diameter of 3.0 mm according to clinic data, which requires that the diameter of a post-expanded stent is usually 1.0–1.1 times the size of the crimped stent. The nominal length of the balloon was set to be 12 mm basing on the operation instructions of stents that the length of the delivery balloon is nominally 2.0 mm longer than the stent. To obtain the geometry of the deflated balloon, we exerted negative pressure on its inner surface, and similar boundary conditions in Gervaso et al. (2008) were referred to create the crimped balloon. The nominal and crimped geometries of the balloon were shown in Figure 1C.

#### Artery and Plaque

The coronary artery is usually curved from patient-specific modeling (Morlacchi et al., 2013; Chiastra et al., 2016). Therefore, the stenotic artery was modeled as a curved cylinder with a central angle of 30° (Wei et al., 2016) and an axisymmetric plaque (Wei et al., 2019). The artery had a curved length of 20.0 mm, an internal diameter of 3.0 mm, and a total wall thickness of 0.5 mm (Pericevic et al., 2009), while the stenotic length and the maximum thickness at the middle cross-section of plaque were 10.0 and 0.6 mm, respectively. The artery consisted of three layers, i.e., intima, media, and adventitia, with a thickness of 0.145, 0.165, and 0.190 mm, respectively (Schiavone et al., 2014). The plaque was established with a base thickness of 0.2 mm (Pant et al., 2012) at both ends of the plaque. The added base was considered: (a) to model a diffused stenosis of the real plaque, (b) to avoid singular meshing if the base thickness was 0.0 mm, and (c) to guarantee the direct contact between the stent and plaque inside the artery (Gervaso et al., 2008; Pant et al., 2012).

### Structural Simulations

The stent expansion inside the curved stenotic artery was performed by the commercial software ABAQUS. The PS-shaped stent, C-shaped stent, and three author-developed stents (C-Rlink, C-Rcrown, and C-Astrut) were made of 316L stainless steel, which had Young's modulus of 196 GPa, Poisson's ratio of 0.3 and yield stress of 375 MPa (Murphy et al., 2003). The XP-shaped stent was constructed by L605 Co-Cr alloy with Young's modulus of 243 GPa, Poisson's ratio of 0.35 and yield stress of 629 MPa (Poncin and Proft, 2003). The semi-compliant balloon was made of single-layered nylon, and modeled as an isotropic, linear-elastic material with Young's modulus of 900 MPa and Poisson's ratio of 0.3 (Gervaso et al., 2008). Artery and plaque were assumed to be incompressible, isotropic, and hyper-elastic (Carew et al., 1968; Karimi et al., 2013), and defined by Ogden and Mooney-Rivlin constitutive equations, respectively. An isotropic hyper-elastic material is often characterized by a polynomial strain energy density function (SEDF) W, which is described as:

$$\begin{array}{l} \text{W}=\overset{\text{N}}{\underset{\text{p}+\text{q}=1}{\sum }}{\text{C}}_{\text{pq}}{\left({\bar{\text{I}}}_{1}-3\right)}^{\text{p}}{\left({\bar{\text{I}}}_{2}-3\right)}^{\text{q}}+\overset{\text{N}}{\underset{\text{p}=1}{\sum }}\frac{1}{{\text{D}}_{\text{p}}}{\left(\text{J}-1\right)}^{2\text{p}}\\ \;\left(\text{p},\text{q}=0,1,\ldots \text{N};p+\text{q}=1,2,\ldots \text{N}\right), \end{array}$$

where ${\bar{\text{I}}}_{\text{1}}$ and ${\bar{\text{I}}}_{2}$ are the first and second strain invariants, respectively, C_pq_ and D_p_ are material parameters, J = det(F) is total volumetric ratio, in which F is the deformation gradient. The two strain invariants are expressed as:

$$\begin{array}{lll} {\bar{\text{I}}}_{1} & = & \lambda_{1}^{2}+\lambda_{2}^{2}+\lambda_{3}^{2},\\ {\bar{\text{I}}}_{2} & = & \lambda_{1}^{2}\lambda_{2}^{2}+\lambda_{1}^{2}\lambda_{3}^{2}+\lambda_{2}^{2}\lambda_{3}^{2}, \end{array}$$

where λ_m_ (m = 1, 2, 3) is the stretch ratio in three principal directions, and it is defined as a ratio of the current length L_m_ and original length L_m, 0_, i.e., λ_m_ = L_m_/L_m, 0_. The Mooney-Rivlin model is a special case of the SEDF, while the Ogden model can be also considered as a polynomial form in terms of the stretch ratios as its variables instead of the invariants. The second-order Mooney-Rivlin model used for plaque and the Ogden model used for arterial layers are defined as:

Plaque:

$$\begin{array}{l} \text{W}={\text{C}}_{\text{10}}\left({\bar{\text{I}}}_{\text{1}}\text{-3}\right)\text{+}{\text{C}}_{\text{01}}\left({\bar{\text{I}}}_{\text{2}}\text{-3}\right)\text{+}{\text{C}}_{\text{20}}{\left({\bar{\text{I}}}_{\text{1}}\text{-3}\right)}^{\text{2}}\\ \;+{\text{C}}_{\text{11}}\left({\bar{\text{I}}}_{\text{1}}\text{-3}\right)\left({\bar{\text{I}}}_{\text{2}}\text{-3}\right)\text{+}{\text{C}}_{\text{02}}{\left({\bar{\text{I}}}_{\text{1}}\text{-3}\right)}^{\text{2}}, \end{array}$$

Arterial layers:

$$\begin{array}{l} \text{W}=\overset{\text{N}}{\underset{\text{i}=1}{\sum }}\frac{\mu_{\text{i}}}{\alpha_{\text{i}}}\left({\bar{\lambda }}_{1}^{\alpha_{\text{i}}}+{\bar{\lambda }}_{2}^{\alpha_{\text{i}}}+{\bar{\lambda }}_{3}^{\alpha_{\text{i}}}-3\right)+\overset{\text{N}}{\underset{\text{i}=1}{\sum }}\frac{1}{{\text{D}}_{\text{i}}}{\left(\text{J}-1\right)}^{2\text{i}}. \end{array}$$

Material parameters C_pq_ were determined by fitting a centrally lying tensile test curve for human plaques reported by Loree et al. (1994), and polynomial coefficients μ_i_ and α_i_ were fit from the experimental data by Karimi et al. (2014) (see Table 1).

**Table 1:** Material parameters for three arterial layers and plaque.

**Arterial layers (Ogden)**	**μ_1_(MPa)**	**μ_2_**	**μ_3_**	**α_1_**	**α_2_**	**α_3_**
Intima	−7.04	4.23	2.85	24.48	25.00	−7.04
Media	−1.23	0.88	0.45	16.59	16.65	−1.23
Adventitia	−1.28	0.85	0.44	24.63	25.00	−1.28
Plaque (Mooney-Rivlin)	C_10_	C_01_	C_20_	C_11_	C_02_	–
Plaque	0.07508	0.1090	1.2935	−2.5342	2.4119	–

The simulation methods for structural analyses were defined as following: For boundary conditions, three nodes forming an equilateral triangle in the central cross-section of the balloon were constrained in axial and circumferential directions to avoid potential rigid displacements. A pinned constraint (U_r_ = 0; r = 1, 2, 3) was applied on the two ends of the artery, while three nodes in the central cross-section of the stent were constrained to only move in the radial direction. Interfaces between three arterial layers were treated as perfectly bonded, and this was also applicable for the interface between the arterial wall and the plaque. The general contact method with a free-friction property was adopted, and such contact was applied to all of balloon self-contact, balloon-stent, balloon-plaque, and stent-plaque contact pairs. The stents were expanded by inflating the balloon as clinic instruction. A pressure of 1.6 MPa was uniformly imposed on the internal surface of the balloon, which consisted of three distinct phases, i.e., loading (0.03 s), holding (0.02 s), and unloading (0.03 s).

The stents were meshed by first-order incompatible brick elements (C3D8I), which consisted of 11,000–22,000 elements depending on their structures. The balloon was discretized with 12,420 reduced integration membrane elements. Both artery and plaque were meshed by hexahedral elements with reduced integration (C3D8R). The element numbers of the meshed artery and plaque were 85,824 and 64,000, respectively in all simulations. The mesh resolution was determined based on a mesh density study, which ensured no penetration between the stent and the plaque in the stenting process. Abaqus/Explicit was used as the solver, and the structural simulations were modeled as dynamic explicit processes. The time increment was in the order of 10^−8^ s throughout the analysis, which confirmed the stability and validity of the simulations.

### Hemodynamic Simulations

**Figure 2:**
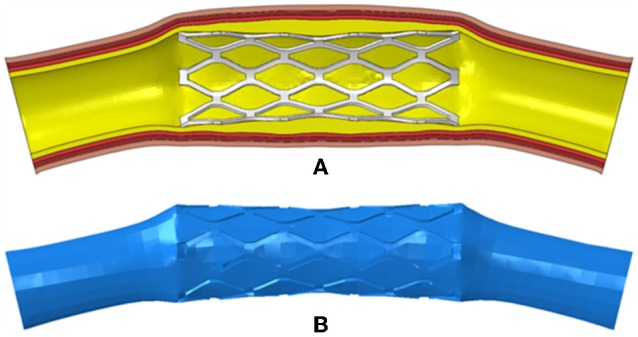
**(A)** The deformed model of the PS-shaped stent, plaque, and artery; **(B)** the fluid domain reconstructed from the deformed model.

To generate the fluid domains after the deployment of the stents, the meshes of the deformed stents and tissues including plaques and arteries were extracted and imported into Hypermesh (Altair Corporation, USA). Then the surface models of the stents and tissues were reconstructed in Hypermesh and imported into Geomagic Studio (Geomagic Corporation, USA). The surface models were repaired in Geomagic Studio, and the complete and closed surface models were generated. Solid models of the deformed stents and tissues were created by using Solidworks, and Boolean operation was performed to obtain the fluid domain (Figure 2).

**Figure 3:**
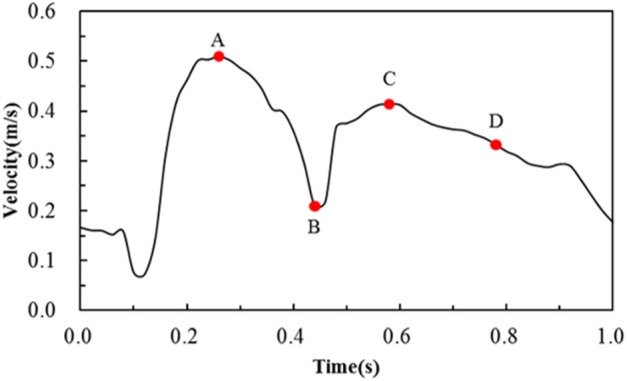
Transient velocity profile employed to simulate pulsatile flow conditions in the human coronary artery. Note that A, B, C, D are four critical time points treated in the following hemodynamic analysis.

The blood was assumed to be incompressible Newtonian fluid with a density of 1,060 kg/m^3^ and a constant viscosity of 0.0035 Pa·s. The fluid domain was meshed by prism and tetrahedron elements, and the number of elements was around 4,500,000 in the six simulations. For boundary conditions, the post-deformed walls in the structural analysis were taken to be rigid with a no-slip condition in the following hemodynamic analysis. The inlet boundary condition for a coronary artery was adopted from literature (Banerjee et al., 2000; Bernad et al., 2012), and the velocity waveform is shown in Figure 3. The outlet flow was supposed to be stable thus a zero pressure was applied to the outlet. The hemodynamic simulations were carried out in ANSYS-Fluent. The coupling between pressure field and velocity field was solved using the Coupled algorithm. The second-order upwind scheme was used as the spatial discretization for the flow governing equations. The time-step size was set as 0.02 s, and a convergence criterion of 10^−4^ was specified.

### Post-processing of Results

Dogboning is a phenomenon that two ends of a stent open larger than the stent center during non-uniform balloon expansion, which has a significant effect on thrombosis and hyperplasia. To evaluate the dogboning effect of the stents, the following equation was used:

$$\begin{array}{l} \text{Dogboning}=\frac{{\text{D}}_{\text{e}}\text{-}{\text{D}}_{\text{m}}}{{\text{D}}_{\text{m}}}\times \text{100}\%, \end{array}$$

where D_e_ is mean diameter at the two ends and D_m_ is mean diameter at the middle cross-section of the post-expanded stent, respectively. Stent recoiling is a “spring back” phenomenon resulting from the elastic-plastic deformation of the stent and the loading pressure on the stent applied by the expanded artery. The stent recoiling was defined as:

$$\begin{array}{l} \text{Recoiling}=\frac{{\text{D}}_{\max}\text{-}{\text{D}}_{\text{unload}}}{{\text{D}}_{\text{unload}}}\times \text{100}\%, \end{array}$$

where D_max_ and D_unload_ are mean diameters of the middle cross-section of the stent in the holding and unloading phases, respectively.

The hemodynamic environment in each deformed model was assessed in terms of the WSS, time-averaged WSS, and OSI. The WSS vector is used to describe flow-induced viscous stress exerted on the luminal surface, and its magnitude is derived from below:

$$\begin{array}{l} |\text{WSS}|=|{\text{n}}_{\text{i}}\cdot \tau_{\text{ij}}|, \end{array}$$

where τ_ij_ is the viscous stress vector and n_i_ is the surface normal vector. The time-averaged WSS (TAWSS) is the averaged magnitude of the WSS over a cardiac cycle, and it is defined as:

$$\begin{array}{l} |\text{TAWSS}|=\frac{1}{\text{T}}\int_{0}^{\text{T}}|\text{WSS}|\text{dt}, \end{array}$$

where T is the time of one cardiac cycle. The OSI is a non-dimensional scalar and is often used to evaluate the oscillatory nature of the blood flows, and it is defined as:

$$\begin{array}{l} OSI=\frac{1}{2}\left(1-\frac{\left|\int_{0}^{\text{T}}\text{WSSdt}\right|}{\int_{0}^{\text{T}}|WSS|\text{dt}}\right). \end{array}$$

It was reported that the low WSS (<0.5 Pa), low TAWSS (<0.5 Pa), and high OSI (>0.1) were associated with cellular proliferation, intimal thickening, and inflammation (Ku, 1997; Malek et al., 1999; LaDisa et al., 2005; Wentzel et al., 2008), and these thresholds were used to evaluate the stenting impact.

## Results

### Structural Analyses

#### The Structural Behavior of Stent

**Figure 4:**
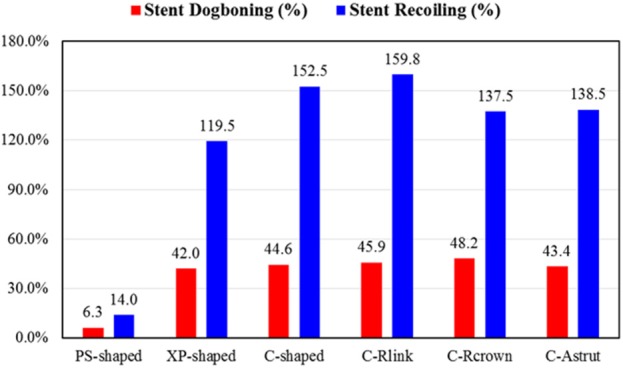
Comparison of stent dogbonings and recoilings for six stent structures.

Figure 4 shows dogbonings and recoilings for the six stents. It was found that the PS-shaped had the least dogboning effect (6.3%), and the other five stents had a similar dogboning effect (around 45.0%). The differences between the author-developed stents (C-Rlink, C-Rcrown, and C-Astrut) and C-shaped were 2.9, 8.1, and −2.8%, respectively, which were relatively small. For stent recoiling, the PS-shaped also had the smallest value (14.0%), followed by XP-shaped < C-Rcrown < C-Astrut < C-shaped < C-Rlink, and this indicates that compared to the C-shaped stent, both C-Rcrown and C-Astrut performed better than the C-shaped in the aspect of stent recoiling.

**Figure 5:**
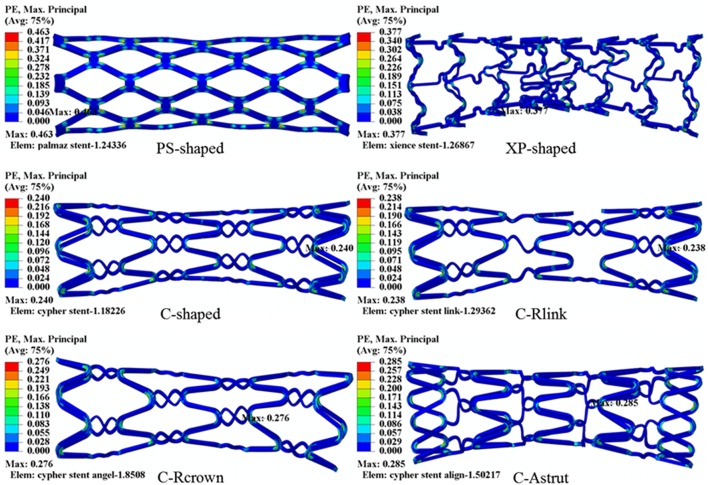
The distributions of maximum principal plastic strain for six stents after implantation.

**Figure 6:**
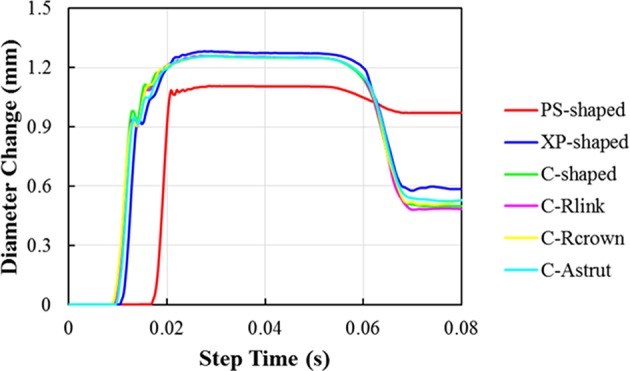
The average diameter changes of six stents during stenting.

The distributions of the maximum principal plastic strain on the stents are shown in Figure 5. It shows that the maximum plastic strain was usually observed located at the curved regions of the struts, and they were in the descending order of PS-shaped > XP-shaped > C-Astrut > C-Rcrown > C-shaped > C-Rlink, which was similar to the order of stent recoiling except the C-Astrut and C-Rcrown. The changes of the stent mean diameters against the step time in the six simulations are shown in Figure 6. Overall, the changes showed a similar trend, and all of them increased sharply during the loading phase, leveled off at a peak value during the holding phase, and declined during the unloading phase. During the holding stage, the PS-shaped had a lower diameter change compared to other stents, but it had the maximum diameter change after stenting, which corresponded to the stent recoiling and the maximum plastic strain. The final diameter change was in the same descending order as the maximum plastic strain, which suggested that a larger plastic strain was favorable to sustain the stent deformation and to result in a larger stent diameter change. Besides, the C-Rcrown and C-Astrut had larger maximum plastic strains as well as greater diameter changes than the C-shaped after stenting, and this indicates that the performance of the author-developed stents was superior to the C-shaped.

#### von Mises Stress on Plaque and Arterial Walls

**Figure 7:**
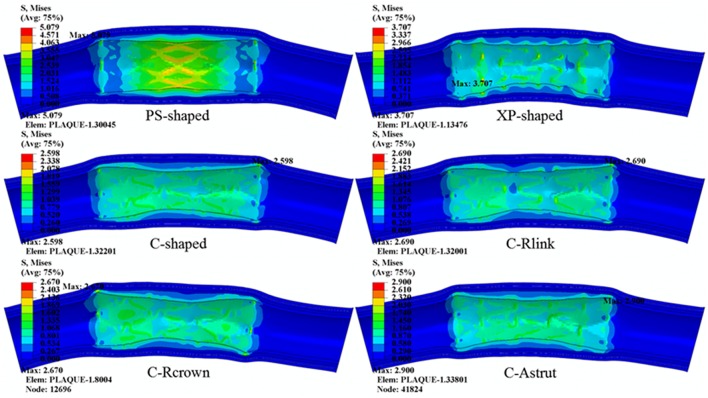
The distributions of von Mises stress on plaque and arterial layers for six stents (units: MPa).

The distributions of von Mises stress on plaque and different arterial layers for all models are shown in Figure 7. It reveals that the maximum stress mainly concentrated on the plaque due to the direct contact between plaque and stent. Besides, stress distributed on the contact area between plaque and stent were over the non-contact area, in particular, the maximum stress of plaque was typically observed in the contact sites near the end of the stent.

The maximum stresses on plaque and arterial layers are shown in Table 2. It was found that the PS-shaped caused the greatest maximum stress on the plaque, and the maximum stresses of plaque within the C-Rlink, C-Rcrown, and C-Astrut models were greater than that within the C-shaped model. For the C-Rcrown and C-Astrut models, this could be easily understood since they had a larger maximum plastic strain and diameter change so as to induce greater stress on the plaque. However, the C-Rlink model also exerted greater stresses on plaque even if it had a smaller maximum plastic strain and diameter change than the C-shaped. The maximum stresses on arterial layers for all models except the XP-shaped were in the descending order of Intima > Media > Adventitia, which suggests the distance between stent and layer plays a role in determining the stress level within it.

**Table 2:** The maximum von Mises stresses on plaque and arterial layers (units: MPa).

**Layers**	**PS-shaped**	**XP-shaped**	**C-shaped**	**C-Rlink**	**C-Rcrown**	**C-Astrut**
Plaque	5.079	3.707	2.598	2.690	2.670	2.900
Intima	1.253	0.274	0.272	0.252	0.271	0.345
Media	0.465	0.279	0.207	0.203	0.233	0.217
Adventitia	0.040	0.087	0.025	0.037	0.063	0.036

### Hemodynamic Analyses

#### Distribution of Wall Shear Stress

**Figure 8:**
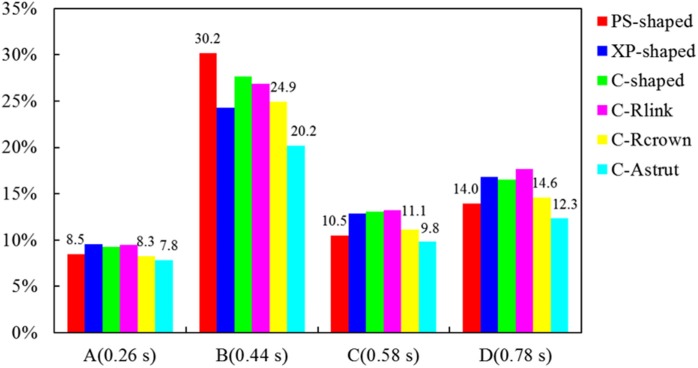
The histogram of area percentages for adverse low WSS (<0.5 Pa) at four critical times over a cardiac cycle.

The area percentages of adverse low WSS (<0.5 Pa) at four critical times in a cardiac cycle (see Figure 3, the four time points A, B, C, and D correspond to 0.26, 0.44, 0.58, and 0.78 s, respectively) are shown in Figure 8. Among the three commercially-shaped stents, the area percentage of low WSS was relatively lower for the PS-shaped during most of the cycle (0.26, 0.58, and 0.78 s), but it was the largest at 0.44 s with a 255% increase when the flow velocity declined greatly from the peak value (0.26 s). For the author-developed stents, the C-Rcrown and C-Astrut showed an improved ability with a smaller area percentage of low WSS compared to that of the C-shaped, while the C-Rlink seemed to have worse performance in most of the cycle except 0.44 s. Also, Figure 8 shows that the C-Astrut had the smallest area percentage of low WSS in the six models, and the area percentage of low WSS for the C-Rcrown was also smaller than the three commercially-shaped stents at 0.26 s, which suggested that using aligned struts structure or reducing the number of strut crowns could improve the performance of the C-shaped in the WSS aspect to some extent so as to make it superior to other commercially-shaped stents.

#### Distribution of Time-Averaged WSS

**Figure 9:**
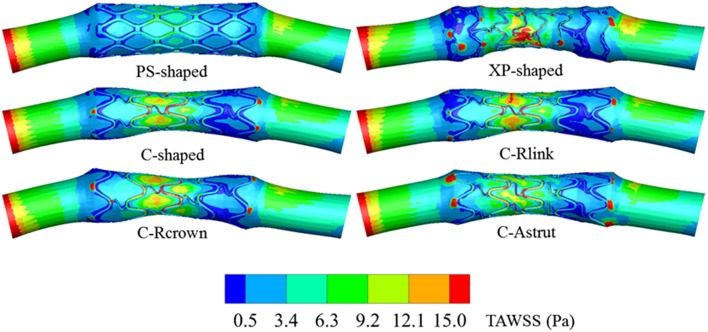
The contours of the TAWSS distributed on the lumen wall.

**Figure 10:**
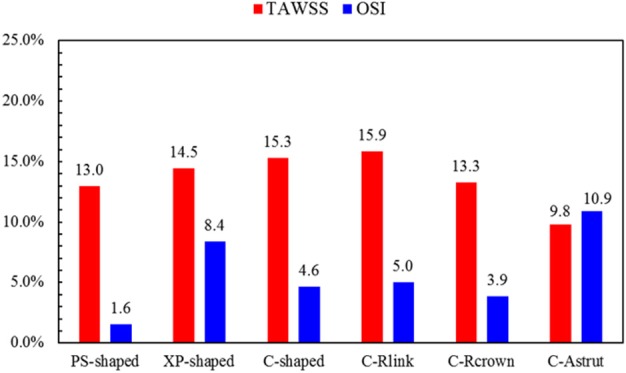
The histogram of area percentages for adverse low TAWSS (<0.5 Pa) and high OSI (>0.1) over a cardiac cycle.

The TAWSS was also introduced throughout the cardiac cycle, and the distributions of the TAWSS are shown in Figure 9. For all models, the lower TAWSS mainly located at sites around struts and links near the ends of the stent, while the higher TAWSS mainly distributed in the middle regions near the center of the stent, which had a smaller cross-section due to the bulged plaque. Moreover, it is shown that the higher TAWSS also located around some curved struts at the ends of the stent and the inlet of the artery, this was mainly due to the effect of higher flow velocity. The area percentages of low TAWSS (<0.5 Pa) for the six models are shown in Figure 10. The PS-shaped still had the smallest area percentage of adverse TAWSS among the three commercially-shaped stents, which suggested a relatively better mechanical performance. Compared to the C-shaped, the area percentage of low TAWSS was 3.8% larger, 13.2%, and 35.8% smaller for the C-Rlink, C-Rcrown, and C-Astrut, respectively. This observation indicates that the C-Rcrown and C-Astrut had a better ability than the C-shaped. In addition, the C-Astrut performed best among the six models, with a reduction of 24.3% in area percentage of low TAWSS when compared to the PS-shaped. Although the C-Rcrown was 2.4% greater than the PS-shaped, the difference was not significant.

#### Distribution of Oscillatory Shear Index

**Figure 11:**
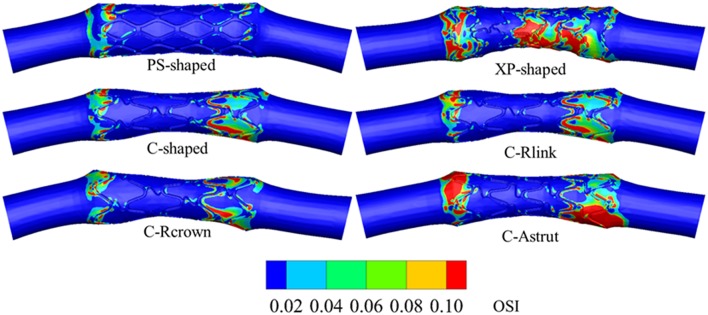
The contours of OSI distributed on the lumen wall.

Figure 11 shows the distributions of OSI for the six models. OSI represents the oscillatory change of the blood flow direction, and its distribution is correlative to that of WSS (Lee et al., 2008; Markl et al., 2010). Figures 9, 11 demonstrated that areas around struts and links near the ends of the stent not only had low WSS, but also had high OSI, which resulted in separation, reversion, and slowing down of the blood flow in these areas, and further accelerated the development of neointimal hyperplasia and ISR. The area percentages of adverse high OSI (>0.1) are shown in Figure 10. The PS-shaped had the smallest percentage of high OSI among the six models. Compared to the C-shaped, the area percentage of C-crown was 16.4% lower while that of C-Rlink and C-Astrut was 8.2 and 134.6% higher, respectively. This showed that only the C-Rcrown had better performance than the C-shaped, while the C-Astrut performed worse.

## Discussions

The results showed that the PS-shaped not only had the least stent dogboning and recoiling, but also had the maximum plastic strain to restrict the deformation of the stent and to result in a larger diameter after stenting. Schiavone et al. (2014) made a comparative study among the Palmaz–Schatz, Cypher, Xience, and Endeavor in the straight plaque and artery model, and concluded the Palmaz–Schatz had the smallest stent recoiling but a higher level of stent dogboning. In their study, various expansion pressures were used for different stents, the current study utilized a crimped balloon with the same expanding pressure to dilate stents. Schiavone et al. (2014) used the straight plaque and artery model in contrast to the curved model in this study. From the viewpoint of plaque and arterial layers, the PS-shaped induced the greatest maximum stress on the plaque, intima, and media among all models (see Table 2), and this corresponded to the maximum plastic strain and diameter change. As the PS-shaped had the largest diameter after stenting, it squeezed the plaque and artery much harder so as to induce the greatest stress on plaque and artery. Bukač et al. (2019) compared the mechanical performance of four commercial stents through fluid-structure interaction analysis, and found that the Cypher-like stent performed best with the smallest deviation in von Mises stress and displacement from the comparisons between stented and non-stented non-plaqued curved coronary artery. In the present study, the C-shaped induced the smallest maximum stress on plaque (see Table 2) as it had smaller maximum plastic strain and diameter change than the PS-shaped and XP-shaped. Differently, Bukač et al. (2019) did not include the plaque in their computational model, which had a great effect on the results. Moreover, they did not study the dogboning and recoiling effects of the stent as well.

Hemodynamically, the PS-shaped displayed a more superior performance among the three commercially-shaped stents during the most part of a cardiac cycle. Compared with the PS-shaped, both the XP-shaped and C-shaped had a curved link structure, thus disrupting the blood flow and resulting in larger regions of adverse low WSS, as well as changing the direction of blood flow and leading to greater areas of high OSI. It has been shown that low and oscillating WSS were related to neointima thickening and atherosclerosis progression, and further led to ISR (Malek et al., 1999; LaDisa et al., 2005), thus the PS-shaped reduced the risk of ISR, and was more favorable than the XP-shaped and C-shaped. Our results were consistent with Balossino et al. (2008), who investigated the effects of different stent designs on local hemodynamics and found the Palmaz-Schatz had a better hemodynamic performance with a lower percentage of low WSS and a more uniform WSS distribution. Chiastra et al. (2016) numerically analyzed the hemodynamic variables by implanting Xience and Nobori stents into image-based patient-specific models, and found that the adverse WSS were less pronounced for Xience in both patients. In this literature, all stents were modeled according to their real dimensions, and the strut of the Xience was thinner than that of the Nobori. However, the thinner struts had a greater positive effect on the hemodynamic environment (Beier et al., 2016). Here, all stent structures shared a thickness to eliminate the influence of strut thickness, thus the XP-shaped had a worse performance compared with other stent structures.

Compared to the C-shaped, the author-developed C-Rlink, C-Rcrown, and C-Astrut had a similar dogboning effect like the C-shaped. Moreover, the C-Rcrown and C-Astrut had better structural performance than the C-shaped in term of stent recoiling, maximum plastic strain and diameter change, which suggested that reducing the number of strut crowns or using aligned struts made it easier for the C-shaped to dilate in the radial direction. The stent was easier to expand in the radial direction suggesting that it had a smaller radial stiffness, which was more favorable from the viewpoint of curing stenosis. Both C-Rcrown and C-Astrut had smaller area percentages of low WSS, low TAWSS than the C-shaped, while only the C-Rcrown had a smaller area percentage of high OSI, which showed that the C-Rcrown generally had a more favorable performance in the hemodynamic aspect. Compared to the C-shaped with six crowns per strut, the C-Rcrown had five crowns per strut which led to bigger angles, and thus resulted in greater gaps between struts. Although the C-Rcrown with fewer crowns reduced the flow alignment between the struts (Gundert et al., 2012a), it had a weaker blocking effect on the flow so as to generate smaller regions of low WSS and high OSI.

From the viewpoint of the combination of structural mechanics and hemodynamics, the PS-shaped stent had better performance than the other two commercially-shaped stents on the basis of the present specific geometrical parameters and evaluation indicators of the stents. However, the real commercial Palmaz-Schatz stent was proved to have a higher risk of the in-stent restenosis than other stents according to clinical studies (Sick et al., 1997; Holmes et al., 2000). This may be because the real commercial Palmaz-Schatz lacks of longitudinal flexibility than other stents (Cho et al., 2007), further inducing vascular endothelial injury and altering the hemodynamic environment. The C-Rlink performed worse than the C-shaped either in structural or in hemodynamic aspect, which suggested that reducing the number of links of the C-shaped cannot improve its ability. The behavior of the C-Astrut was better than the C-shaped except in the OSI aspect, and this indicated that for a stent with aligned struts, it was important to modify the design so as to reduce the area of high OSI. The C-Rcrown generally had better therapeutic effects in both aspects, which suggested that reducing the number of strut crowns of a stent is an effective method to improve the performance of a stent.

In summary, the novelty of this study is that both structural mechanical and hemodynamic analyses were performed on a three-layered curved artery with plaque, which is different from most previous studies in the literature. For instance, Capelli et al. (2009) only evaluated the mechanical effects of five different balloon-expandable stents in a coronary artery through structural mechanical analyses, and the arterial model in this work was treated as a three-layered straight cylinder with no stenosis. Mortier et al. (2011) compared the stent strut apposition of six different stent designs by quantifying the stent induced vessel wall stresses, and similar to Capelli et al. (2009), they only assessed the performance from the structural mechanical aspect. Besides, Conway et al. (2012), Pant et al. (2012), Francesco et al. (2014), and Mortier et al. (2014) also performed structural analyses. Although some of them adopted more complicated arterial models, the hemodynamic analyses were ignored in these studies. On the other hand, Chen et al. (2017) only performed computational fluid dynamics study of different stent models inside curved coronary arteries, but the fluid domain was constructed basing on ideally non-deformed stent and artery. Whereas, our study considered the deformed geometry of both the stent and tissue (including plaque and artery), which may greatly influence the fluid field.

Limitations of the current study included that both plaque and artery were assumed to be incompressible, isotropic, and hyper-elastic materials in the structural analyses in contrast to the visco-elastic or visco-hyper-elastic properties in reality. However, viscoelastic data of the tissues is not available, especially for plaque. The post-deformed wall was assumed to be rigid in the hemodynamic analyses. It was reported that the near-wall quantities such as the WSS of the assumed rigid wall had similar trends like that of the compliant wall, and such simplification was reasonable for hemodynamic simulations (Chiastra et al., 2014). Besides, in reality, the ends of the deflated balloon are rounded, while they were sharp in this study. This may slightly influence the results when the inflated sharp-ended balloon touches the walls. Moreover, a pressure of 1.6 MPa was exerted on the inner surface of the balloon in all models, and this was slightly greater than the nominal pressure specified for commercial stents. Although once the nominal diameter of the balloon is reached, the transmission of the pressure increase to the stent and tissue is very limited (Gervaso et al., 2008), and the pressure adopted in this study might influence the stent performance and the stress on the tissue as well. Despite these limitations, the present work demonstrated the performance of three commercially-shaped stents and three author-developed stents in curved arteries, and might provide a useful method to evaluate the suitability of a stent for a patient with a curved and stenotic coronary artery in the future.

## Conclusions

In this study, three-dimensional structural and hemodynamic simulations for six different stents were performed to study the effects of stents on their performances after stenting. The PS-shaped stent was found to have a better performance than the other two commercially-shaped stents in both structural and hemodynamic aspects. Compared to the C-shaped stent, the author-developed C-Rcrown performed better, which suggested that the performance of the C-shaped stent could be improved by reducing the number of its strut crowns. This study will be helpful to the clinical choice of different commercial stents according to the requirements of the patient-specific model, and may also guide the stent design.

## Data Availability Statement

All datasets generated for this study are included in the manuscript.

## Author Contributions

LW, QC, and ZL presented the concept of the work. LW performed the FEA and CFD computations and drafted the manuscript. LW and QC analyzed the data. HL provided suggestion and editing assistance. QC and ZL critically revised the manuscript. All the authors approved the final version and made substantial contributions to this work.

### Conflict of Interest

The authors declare that the research was conducted in the absence of any commercial or financial relationships that could be construed as a potential conflict of interest.
